# Oral Health as a Predictor of Physical Frailty among Rural Community-Dwelling Elderly in an Agricultural County of Taiwan: A Cross-Sectional Study

**DOI:** 10.3390/ijerph18189805

**Published:** 2021-09-17

**Authors:** Ya-Wen Kuo, Mei-Yen Chen, Li-Ching Chang, Jiann-Der Lee

**Affiliations:** 1 Department of Nursing, College of Nursing, Chang Gung University of Science and Technology, No. 2, Sec. W., Jiapu Rd., Puzi City 613, Chiayi, Taiwan; ywkuo@mail.cgust.edu.tw (Y.-W.K.); meiyen@mail.cgust.edu.tw (M.-Y.C.); 2 Department of Nursing, Chang Gung University, No. 259, Wenhua 1st Rd., Guishan Dist., Taoyuan 333, Taiwan; 3 Department of Dentistry, Chang Gung Memorial Hospital Chiayi Branch, No. 6, West Sec., Jiapu Road, Puzi City 613, Chiayi, Taiwan; lcc1891@cgmh.org.tw; 4 Department of Neurology, Chang Gung Memorial Hospital Chiayi Branch, No. 6, West Sec., Jiapu Road, Puzi City 613, Chiayi, Taiwan; 5 Department of Medicine, Chang Gung University, No. 259, Wenhua 1st Rd., Guishan Dist., Taoyuan 333, Taiwan

**Keywords:** oral health, physical frailty, rural elderly, agricultural

## Abstract

We conducted a cross-sectional study to clarify the relationship between oral health and physical frailty (PF). A sample of 903 community-dwelling individuals aged ≥ 65 years were enrolled from random communities in Chiayi County. The self-perceived oral health (SPOH) and oral health assessment tool (OHAT), which consists of eight items, was used for the evaluation of their oral health status. PF was assessed based on the Study of Osteoporotic Fracture index. Overall, 14.6% of the participants had PF. In an adjusted model, restricted food types (odds ratio (OR) = 1.59, 95% confidence interval (CI): 1.2–2.09, *p* = 0.001), self-reported dental status (OR = 1.61, 95% CI: 1.2–2.15, *p* = 0.001), number of teeth (OR = 0.98, 95% CI: 0.96–0.99, *p* = 0.006), frequency of tooth cleaning (OR = 0.83, 95% CI: 0.68–1.0, *p* = 0.049), OHAT score (OR = 1.09, 95% CI: 1.02–1.17, *p* < 0.017), and saliva items of OHAT (OR = 1.52, 95% CI: 1.11–2.1, *p* = 0.010) were significantly associated with PF. SPOH is a crucial indicator of PF; longitudinal analyses are necessary to understand the underlying pathway of risk factors for frailty onset.

## 1. Introduction

Population aging is a global problem, and frailty in older people is becoming increasingly prevalent, highlighting the need for the early identification of those at frailty risk. In Taiwan, by the end of June 2021, >16% of the population was aged ≥ 65 years, and Chiayi County, with a predominantly agricultural economy, is the part of Taiwan with the largest proportion of older residents: 20.69% of the county’s population [1]. Frailty is typically defined as an aging-related syndrome in which at least three of the following criteria are present: unintentional weight loss, self-reported exhaustion, weakness, slow walking speed, and low physical activity [2].

Frailty is also a syndrome of physiological decline characterized by the Study of Osteoporotic Fractures (SOF) index as the presence of at least two of the following components: weight loss, exhaustion, and low mobility [3]. The prevalence rates of frailty and prefrailty, respectively, among people aged ≥ 65 years are 11.2% and 50.1% in Taiwan [4]. Frailty and low activity levels can cause numerous physiological problems that are also related to the increased risk of falls, disability, hospitalization, and death [5]. A study revealed that frailty in elderly adults can lead to limitations in activities of daily living (odds ratio (OR) = 2.3), increased falls (OR = 1.6), emergency service requirements (OR = 1.9), and reduced quality of life (OR = 3.4) [6].

Oral health acts as a mirror that can reflect problems in the physical system, and oral health is regarded as a part of overall health [7]. In Taiwan, medical expenditure statistics of the National Health Insurance system indicate that oral and salivary gland diseases accounted for the second-highest medical expenses in 2019, and older people was the group claiming such expenses the most [8]. Poor oral hygiene leads to the accumulation of numerous bacteria in the oral cavity, resulting in dental caries, periodontal disease, and chewing disorders [9]. The World Health Organization listed periodontal disease as a global chronic disease in 2019, and oral diseases are the most common noncommunicable diseases that are often ignored by people [10]. If oral problems caused by poor oral health are not detected and treated early, inflammation may gradually affect the tooth-supporting structures and eventually lead to tooth loss [7].

Frail elderly people tend to have poorer oral hygiene than their nonfrail counterparts [11]. A study of elderly people in Mexico revealed that, for every additional intact tooth, the probability of frailty decreased by 5% [12]. Tooth loss may be the end result of poor oral health; a missing tooth further affects tooth function, which reduces the chewing ability and nutrient intake, eventually leading to chronic diseases [13]. Poor oral health, or even oral frailty, increases physical frailty (PF), sarcopenia, and disability, which are significantly related to the risk of death [14]. Oral health problems, including dental pain and dry mouth, are most commonly reported by community-dwelling elderly people [15]. Patients with dental pain and chewing problems are significantly more likely to exhibit PF [16]. Poor oral health is associated with poor food intake and PF, and every additional oral problem increases the index of PF [17].

Frail elderly people have significantly fewer teeth, lower biting abilities, and poorer masseter muscle thickness than nonfrail elderly people, and older age, depression, a low skeletal muscle mass index, a low Mini-Mental State Examination score, high blood pressure, diabetes, high levels of albumin and triglycerides, and low oral function are significantly related to PF [18]. Studies have examined tooth loss due to poor oral health [12,19], but the relationship between the number of teeth and frailty remains uncertain [18,20,21,22]. Research has highlighted that various oral health problems can affect PF [17,18], but the relationship between self-perceived oral health (SPOH) and the SOF frailty criteria of elderly people in rural agricultural communities is unexplored.

This study investigated the distribution of the SPOH and oral health assessment tool (OHAT) score in a population with diverse frailty levels in a rural community to assess its correlation with the associated parameters (participant characteristics). A PF assessment tool with SOF based on a straightforward, rapid, and frequent person-centered care approach is critical for elderly people in rural agricultural communities. Therefore, we examined the relationship between the SPOH and SOF frailty criteria to provide an empirical result for the early detection of poor oral health and PF in elderly people living in rural areas.

## 2. Materials and Methods

A cross-sectional study was conducted in 25 communities from August 2019 to July 2020. We included community-dwelling elderly people living in Chiayi County, which is a rural area with the highest proportion of the elderly population in Taiwan. In 2021, the proportion of elderly people in this county reached 20.69%. Chiayi County has 18 towns and cities, which are major agricultural areas, with a total population of 499,481. This research randomly selected communities through a lottery, and after approval from the person in charge of the community health center, we recruited elderly people for participants in this study. The inclusion criteria were (1) age ≥ 65 years and (2) ability to communicate in Taiwanese or Mandarin. The exclusion criteria were individuals who (1) cannot cooperate because of mental disorders and (2) had decreased cognitive function (Short Portable Mental State Questionnaire (SPMSQ) scale < 6) [23]. This study followed the standards of the Declaration of Helsinki and was approved by the Institutional Review Board of National Cheng Kung University Human Research Ethics Committee (No. 108-033). All participants took part voluntarily and were included in the study after providing informed consent; moreover, the data included in this study were recorded with serial numbers and are anonymous.

### 2.1. Participant Characteristics

A self-administered questionnaire was used in this study, which assessed the following characteristics: age; gender; education level; body mass index (BMI); hospitalized within 1 year; chronic diseases; self-reported health status (good, fair, or poor); and cognitive function. SPOH was assessed with the question: Do you have restriction of food intake type, loose teeth, toothache, halitosis, periodontal disease, tooth decay, dentures, and regular teeth scaling? to which the respondents answered “yes” or “no”. The self-reported dental status was assessed with one question: How do you perceive your dental status to be? Three response options were provided: “poor”, “fair”, or “good”.

The number of remaining teeth and frequency of teeth cleaning were calculated in this study. The BMI formula kg/m^2^ was used, where kg is a person’s weight in kilograms, and m^2^ is their height in meters squared. Cognitive function was measured using the SPMSQ scale, which contains six dimensions: consciousness, memory, orientation, attention, thinking, and general knowledge; the retest reliability of this scale is 0.82–0.83; the full score is 10, with scores of 8–10, 6–7, 3–5, and ≤2 indicating normal cognitive functioning and mild, moderate, and severe cognitive dysfunction, respectively [23].

### 2.2. Physical Frailty

The SOF scale was used to assess the PF of elderly people. The SOF scale is a tool of quick, simple operation and is not limited to the clinical field for PF assessments of elderly people. Furthermore, the SOF scale can significantly predict PF [24]. Therefore, the SOF scale is used to assess the PF of elderly people in community care centers by the Ministry of Health and Welfare of Taiwan. The SOF index was determined according to the presence of at least two of the following three components: (1) weight loss, a weight loss of ≥5% during the preceding year (one point); (2) lower limb function, inability to rise from a chair five times without using the arms (one point); and (3) poor energy, a reply of “no” to the question, “Do you feel full of energy?” (one point). SOF scale scores range from 0 to 3. If at least two of the three criteria are met, it indicates frailty; if one of the three criteria is met, it indicates prefrailty or intermediate frailty; if none of the criteria are met, it indicates nonfrailty [25].

### 2.3. Oral Health Assessments

The oral health assessment is based on the OHAT score. The OHAT is an organized, efficient method for evaluating oral health and has been verified as an assessment tool suitable for nondental healthcare professionals to assess the oral health of elderly people [26]. It is used to assess the oral health of elderly people in community care centers by the Ministry of Health and Welfare of Taiwan. OHAT has eight items—namely, the lips, tongue, gums and tissues, saliva, natural teeth, dentures, oral cleanliness, and toothaches. Data collection was conducted by the same trained researcher. Data regarding the lips, tongue, tongue, gums, saliva, natural teeth, and dentures were collected based on direct observations. Oral cleaning was determined through observation and plaque detection agent use. Toothaches were determined through inquiry and observation. This tool employs a 3-point (0, 1, and 2) scale for the eight items. Each category was rated with the values 0 = healthy, 1 = changes, or 2 = unhealthy; the final score was the sum of the scores from the eight categories, ranging from 0 (very healthy) to 16 (very unhealthy); the higher the score, the poorer the oral health condition. The OHAT has favorable reliability and validity; the overall intraclass correlation coefficient for the total OHAT score was 0.78, and all the results were statistically significant. For individual items, the inter-rater reliability ranged from 74.4% agreement for oral cleanliness to 93.9% for dental pain and 96.6% for a referral to the dentist. The validity analyses of the OHAT categories and examination findings indicated highly significant correlations and strong agreements [27].

### 2.4. Statistics

The data were analyzed with SPSS software ver. 26.0 (IBM, Armonk, NY, USA); descriptive variables were presented as the mean, standard deviation, and percentage; continuous variables were presented as the mean ± standard deviation; and categorical variables were presented as percentages. The inferential statistics used were chi-square, one-way analysis of variance, and logistic regression analysis. Logistic regression was performed to generate ORs adjusted for the variables of the study participants. A *p*-value of <0.05 was considered statistically significant.

## 3. Results

In total, 952 elderly people were included in this study. After excluding invalid questionnaires with incomplete answers, the final sample size was 903 (615 women; mean age: 77.4 ± 7.1 years), and the effective recovery rate was 94.9%. The differences in the demographic data between those with no frailty (*n* = 471, 52.2%), prefrailty (*n* = 300, 33.2%), and frailty (*n* = 132, 14.6%) were compared. Age; gender; education level; hospitalization within 1 year; chronic diseases (diabetes, heart disease, and arthritis); SPMSQ; and self-reported health conditions of elderly people at different frailty levels exhibited significant differences (*p* < 0.05). Of the elderly people, 79.2% had an educational attainment of elementary school or below. Elderly people with diabetes, heart disease, and arthritis comprised >50% of the population with prefrailty and frailty. Cognitive dysfunction with the SPMSQ was observed in 62.6% of the abnormal population with prefrailty and frailty. Fair or poor health status were reported by 52.1% of the elderly population with prefrailty and frailty (Table 1).

Elderly people with various frailty levels were compared in terms of their perceived oral health. The results indicated that the restriction of food intake type (*p* < 0.001), loose teeth (*p* = 0.021), self-reported oral health status (*p* = 0.001), number of teeth (*p* < 0.001), frequency of dental cleaning (*p* = 0.012), and regular teeth scaling (*p* = 0.015) were significantly different (Table 2).

We compared the total OHAT score and those of its subdomains for elderly people with different frailty levels. The results revealed significant differences in the saliva (*p* = 0.008), natural teeth (*p* = 0.023), and toothache (*p* = 0.007) subdomains and the total mean score of the OHAT (*p* < 0.001; Table 3).

A logistic regression analysis revealed that the restricted food type (OR = 1.83, 95% confidence interval (CI): 1.4–2.38, *p* < 0.001), self-reported dental status (OR = 1.66, 95% CI: 1.25–2.19, *p* = 0.001), number of teeth (OR = 0.97, 95% CI: 0.95–0.98, *p* < 0.001), frequency of tooth cleaning (OR = 0.79, 95% CI: 0.66–0.94, *p* = 0.008), regular teeth scaling (OR = 0.58, 95% CI: 0.37–0.92, *p* = 0.019), total OHAT score (OR = 1.15, 95% CI: 1.08–1.23, *p* < 0.001), and OHAT subdomains for saliva (OR = 1.54, 95% CI: 1.13–2.09, *p* = 0.006) and natural teeth (OR = 0.74, 95% CI: 0.57–0.97, *p* = 0.026) could predict PF in a crude model. In the adjusted model, restricted food type (OR = 1.59, 95% CI: 1.2–2.09, *p* = 0.001), self-reported dental status (OR = 1.61, 95% CI: 1.2–2.15, *p* = 0.001), number of teeth (OR = 0.98, 95% CI: 0.96–0.99, *p* = 0.006), frequency of teeth cleaning (OR = 0.83, 95% CI: 0.68–1.0, *p* = 0.049), total OHAT score (OR = 1.09, 95% CI: 1.02–1.17, *p* < 0.017), and the saliva OHAT subdomain (OR = 1.52, 95% CI: 1.11–2.1, *p* = 0.010) were significantly associated with PF (Table 4).

## 4. Discussion

In this study, we explored the association between SPOH and PF. In addition, we examined the correlation of the characteristics of the study participants, OHAT, OHAT subdomains, and PF with the predictors of PF. In this study, a PF risk was associated with the restricted food type, self-reported dental health, number of teeth, frequency of tooth cleaning, OHAT score, and saliva items of the OHAT subdomain, and these variables could predict PF.

Oral diseases are closely related to systemic diseases, and poor oral health intensifies the effect of systemic diseases [28]. We observed an association between the PF risk and poor oral health, which included restricted food type and number of teeth. Poor oral health is associated with oral frailty, as determined by six factors—namely, articulatory oral motor skill, chewing ability, tongue pressure, subjective difficulties in eating and swallowing, and number of remaining teeth [14]. Oral frailty is defined as poor oral status in at least three of the six measures, and it was significantly associated with a 2.4- and 2.2-fold increased risk in PF and mortality, respectively, among community-dwelling elderly people [14]. Our study revealed that the average number of teeth of rural elderly people is 8.9, and the number of teeth decreases as the degree of frailty increases; the number of teeth can be a predictor of PF. The results of our study are similar to those of a study reporting that PF is associated with having fewer teeth (≤20 teeth) among older adults [29]. Another study indicated negative associations between oral frailty and food satisfaction; comprehensive oral function not only concerns the number of teeth but, also, the satisfaction of elderly people in terms of food intake, which can support optimal nutrition [30]. Oral problems in terms of restricted food type and self-reported dental status may increase the awareness among elderly people and help them easily understand the risks of PF.

Oral health–related behaviors and oral frailty concepts are included in the Oral Frailty Index-8, an eight-item screening questionnaire that assesses oral health-related behaviors in terms of the number of times teeth are brushed per day; teeth brushing less than three times a day indicates poor oral health [31]. In the present study, the rural elderly respondents reported brushing their teeth on average 1.99 times a day; the frequency of tooth cleaning differed significantly between the nonfrail and frail groups and can be a predictor of PF. Frail elderly people require guidance and support regarding oral cleaning habits, which can benefit oral health, and oral health should be included in the daily care plan of home care for elderly people [11].

In the present study, the OHAT score and saliva items of the OHAT subdomain score were predictors of frailty according to the SOF frailty criteria in the frail elderly of the rural community. The pilot study established a relationship between the OHAT and Fried frailty criteria in a population of frail elderly people; however, the distribution of various items composing the OHAT score was not explored [32]. The role of saliva in maintaining favorable oral health has received little attention in most populations; the unstimulated low flow rate of saliva not only increases an individual’s susceptibility to xerostomia but also greatly delays the clearance of food from the mouth [33]. Saliva is essential to oral health, because if saliva is lacking or unhealthy, it will lose its protective effect, increasing the susceptibility of teeth to various diseases [33]. For the elderly population, oral health care should receive the maximum attention to prevent xerostomia, and related dry mouth assessment and care strategies should be prioritized in the care process.

Many community-dwelling elderly people have oral hypofunction, which is significantly associated with frailty [34]. In this study, 33.2% and 14.6% of the elderly people exhibited prefrailty and frailty, respectively, and 65.7% of the elderly self-reported a fair or poor dental status. A study in Japan revealed that the PF risk (OR = 2.40, *p* = 0.012) is associated with oral frailty [35], and in the rural community-dwelling older adults, the oral frailty and hypofunction rates were 22.5% and 43.6%, respectively [36]. Thus, elderly people in the rural communities of Taiwan have oral frailty that meets at least three of the six oral frailty indicators, highlighting the need for the improved screening, care, and education of elderly people in the future.

Frailty is a common consequence of aging [37]. In the present study, an increase in age was associated with a gradual increase in the extent of PF. This result indicates that the elderly people in rural communities in Taiwan experience considerable frailty due to aging. Therefore, evidence-based dentistry was important in making decisions about the oral health of the elderly [38]. Policies and strategies must be strengthened to prevent oral frailty and PF, and people’s health must receive attention at an earlier stage of aging to ensure healthy aging. In addition, 79.2% of the elderly people in the investigated agricultural county had an education level of elementary school or below. Elderly people in rural communities should receive education regarding regular oral health assessments, use the correct teeth-cleaning tools to improve gingival health [39], and early dental referral and care should be employed in this population to reduce the likelihood of PF. This study had three main limitations. First, the participants were randomly selected from rural communities, but they volunteered to be included in this study; subsequently, 49 were excluded because of incomplete information regarding personal factors, which may have resulted in a selection bias. Second, the sample represented a specific rural community in an agricultural county; thus, the results cannot be generalized. Third, this was a cross-sectional study, which precluded any meaningful discussions of causality.

## 5. Conclusions

Regardless of the above limitations, the findings of this study revealed that perceived oral health, along with restricted food type, self-reported dental status, number of teeth, frequency of tooth cleaning, OHAT score, and saliva items of the OHAT subdomain score, were associated with PF indicators of the SOF scale and could predict PF. It is essential to initiate an oral health-promoting program as a routine assessment and the education of elderly people, e.g., enhancing the benefits of good oral hygiene, self-perceived oral health education, and reducing the barriers of adopting regular oral function exercise through the empowerment strategy. Our findings suggest that oral health should be promoted earlier in health policies, and it should be regarded as a critical factor for achieving healthy aging. SPOH may be crucial indicators of PF onset, but longitudinal studies are necessary to understand whether a causal relationship exists.

## Figures and Tables

**Table 1:** Characteristics of the study participants.

Variables	Overall (*n* = 903)	Nonfrail (*n* = 471)	Prefrailty (*n* = 300)	Frailty (*n* = 132)	*p-*Value
Age, years, mean (SD)	77.4 (7.1)	76.1 (6.7)	78.2 (7.5)	80.1 (6.8)	<0.001 ^b^
Gender, female, *n* (%)	615 (68)	311 (66)	202 (67)	102 (77)	0.047 ^a^
Body mass index, kg/m^2^, mean (SD)	24.1 (3.8)	24.2 (3.8)	24.2 (3.7)	23.4 (3.7)	0.069 ^b^
Education level, *n* (%)					<0.001 ^a^
Elementary school and below	715 (79.2)	350 (74)	248 (83)	117 (89)	
Junior high school and above	188 (21)	121 (26)	52 (17)	15 (11)	
Hospitalization within 1 year (yes), %	104 (11.5)	39 (8.3)	40 (13.3)	25 (18.9)	0.002 ^a^
Chronic disease					
Hypertension (yes), %	436 (48.3)	234 (49.7)	136 (45.3)	66 (50)	0.456 ^a^
Diabetes mellitus (yes), %	208 (23)	91 (19.3)	76 (25.3)	41 (31.1)	0.009 ^a^
Heart disease (yes), %	145 (16.1)	63 (13.4)	51 (17)	31 (23.5)	0.017 ^a^
Stroke (yes), %	27 (3)	11 (2.3)	12 (4)	4 (3)	0.417 ^a^
Arthritis (yes), %	133 (14.7)	63 (13.4)	35 (11.7)	35 (26.5)	<0.001 ^a^
SPMSQ					<0.001 ^a^
Normal, %	676 (74.9)	386 (82)	208 (69.3)	82 (62.1)	
Abnormal, %	227 (25.1)	85 (18)	92 (3.7)	50 (37.9)	
Self-reported health status					<0.001 ^a^
Good, %	310 (34.3)	187 (39.7)	89 (29.7)	34 (25.8)	
Fair or poor, %	593 (65.7)	284 (6.3)	211 (7.3)	98 (74.2)	

SD, standard deviation; SPMSQ, Short Portable Mental State Questionnaire. Mean ± SD for continuous variables, and percentages for categorical variables. ^a^ χ^2^ test for proportions and nominal variables. ^b^ Based on a one-way analysis of variance.

**Table 2:** Distribution of the participants based on self-perceived oral health.

Variables	Overall (*n* = 903)	Nonfrail (*n* = 471)	Prefrailty (*n* = 300)	Frailty (*n* = 132)	*p-*Value
Restricted food intake type (yes), %	486 (53.8)	220 (46.7)	171 (57)	95 (72)	<0.001 ^a^
Loose teeth (yes), *n* (%)	169 (18.7)	84 (17.8)	49 (16.3)	36 (27.3)	0.021 ^a^
Toothache (yes), *n* (%)	618 (68.4)	311 (66)	208 (69.3)	99 (75)	0.135 ^a^
Halitosis (yes), *n* (%)	242 (26.8)	114 (24.2)	86 (28.7)	42 (31.8)	0.146 ^a^
Periodontal disease (yes), *n* (%)	197 (21.8)	94 (20)	69 (23)	34 (25.8)	0.301 ^a^
Tooth decay (yes), *n* (%)	242 (26.8)	129 (27.4)	74 (24.7)	39 (29.5)	0.526 ^a^
Denture (yes), *n* (%)	787 (87.2)	404 (85.8)	264 (88)	119 (9.2)	0.359 ^a^
Regular teeth scaling (yes), *n* (%)	89 (9.9)	57 (12.1)	27 (9)	5 (3.8)	0.015 ^a^
Self-reported dental status, *n* (%)					0.001 ^a^
Good	310 (34.3)	187 (39.7)	89 (29.7)	34 (25.8)	
Fair/poor	593 (65.7)	284 (6.3)	211 (7.3)	98 (74.2)	
Number of remaining teeth, mean (SD)	8.9 (9.9)	10.5 (10.5)	7.7 (9.3)	5.8 (7.9)	<0.001 ^b^
Frequency of tooth cleaning, mean (SD)	1.99 (0.83)	2.07 (0.86)	1.92 (0.8)	1.86 (0.8)	0.012 ^b^

Mean ± SD for continuous variables, and percentages for categorical variables. ^a^ χ^2^ test for proportions variables. ^b^ Based on a one-way analysis of variance.

**Table 3:** OHAT and subdomains for elderly people with different frailty levels.

Variables	Overall (*n* = 903)	Nonfrail (*n* = 471)	Prefrailty (*n* = 300)	Frailty (*n* = 132)	*p-*Value
Lip, *n*%					0.059 ^a^
Normal	419 (46.4)	226 (48)	142 (47.3)	51 (38.6)	
Change	479 (53)	244 (51.8)	154 (51.3)	81 (61.4)	
Unhealthy	5 (0.6)	1 (0.2)	4 (1.3)	0 (0)	
Tongue, *n*%					0.408 ^a^
Normal	551 (61)	301 (63.9)	177 (59)	73 (55.3)	
Change	346 (38.3)	167 (35.5)	121 (4.3)	58 (43.9)	
Unhealthy	6 (0.7)	3 (0.6)	2 (0.7)	1 (0.8)	
Gum and tissue, *n*%					0.720 ^a^
Normal	681 (75.4)	358 (76)	229 (76.3)	94 (71.2)	
Change	220 (24.4)	112 (23.8)	70 (23.3)	38 (28.8)	
Unhealthy	2 (0.2)	1 (0.2)	1 (0.3)	0 (0)	
Saliva, *n*%					0.008 ^a^
Normal	685 (75.9)	375 (79.6)	219 (73)	91 (68.9)	
Change	217 (24)	96 (2.4)	81 (27)	40 (3.3)	
Unhealthy	1 (0.1)	0 (0)	0 (0)	1 (0.8)	
Natural teeth, *n*%					0.023 ^a^
Normal	365 (4.4)	174 (36.9)	132 (44)	59 (44.7)	
Change	293 (32.4)	176 (37.4)	80 (26.7)	37 (28)	
Unhealthy	245 (27.1)	121 (25.7)	88 (29.3)	36 (27.3)	
Denture, *n*%					0.223 ^a^
Normal	338 (37.4)	189 (4.1)	111 (37)	38 (28.8)	
Change	521 (57.7)	262 (55.6)	171 (57)	88 (66.7)	
Unhealthy	44 (4.9)	20 (4.2)	18 (6)	6 (4.5)	
Oral cleanliness status, *n*%					0.157 ^a^
Normal	24 (2.7)	15 (3.2)	8 (2.7)	1 (0.8)	
Change	861 (95.3)	451 (95.8)	283 (94.3)	127 (96.2)	
Unhealthy	18 (2)	5 (1.1)	9 (3)	4 (3)	
Dental pain, *n*%					0.007 ^a^
Normal	782 (86.6)	414 (87.9)	265 (88.3)	103 (78)	
Change	0 (0)	0 (0)	0 (0)	0 (0)	
Unhealthy	121 (13.4)	57 (12.1)	35 (11.7)	29 (22)	
OHAT, mean (SD)	4.5 (2.0)	4.3 (2.0)	4.6 (2.1)	5.5 (1.8)	<0.001 ^b^

OHAT, oral health assessment tool. Mean ± SD for continuous variables, and percentages. For categorical variables. ^a^ χ^2^ test for proportions variables. ^b^ Based on a one-way analysis of variance.

**Table 4:** Relationship between oral health and physical frailty.

	Crude Model			Adjusted Model		
	OR	95% CI	*p*	OR	95% CI	*p*
Self-perceived oral health						
Restricted food type	1.83	1.4–2.38	<0.001	1.59	1.2–2.09	0.001
Loose teeth	1.13	0.81–1.58	0.479	1.16	0.81–1.64	0.418
Self-reported dental status	1.66	1.25–2.19	<0.001	1.61	1.2–2.15	0.001
Number of teeth	0.97	0.95–0.98	<0.001	0.98	0.96–0.99	0.006
Frequency of tooth cleaning	0.79	0.66–0.94	0.008	0.83	0.68–1.0	0.049
Teeth scaling regularly	0.58	0.37–0.92	0.019	0.79	0.49–1.27	0.327
OHAT score	1.15	1.08–1.23	<0.001	1.09	1.02–1.17	0.017
OHAT subdomains						
Saliva	1.54	1.13–2.09	0.006	1.52	1.11–2.1	0.010
Natural teeth	0.74	0.57–0.97	0.026	0.86	0.65–1.14	0.305
Toothache	1.26	0.86–1.85	0.233	1.20	0.8–1.78	0.375

The adjusted model was adjusted for age, gender, education, hospitalization, chronic disease, and SPMSQ. OR, odds ratio; CI, confidence interval.

## Data Availability

The data that support the findings of this study can be obtained from the corresponding author upon reasonable request.

## References

[1] Department of Household Registration, Ministry of the Interior (2021). Demographic Data. https://doi.org/https://www.ris.gov.tw/app/portal/346.

[2] Fried L.P., Tangen C.M., Walston J.D., Newman A.B., Hirsch C., Gottdiener J.S., Seeman T.E., Tracy R.P., Kop W.J., Burke G.L. (2001). Frailty in Older Adults: Evidence for a Phenotype. J. Gerontol. Ser. A Biol. Sci. Med. Sci..

[3] Si H., Jin Y., Qiao X., Tian X., Liu X., Wang C. (2021). Comparison of 6 frailty screening tools in diagnostic properties among Chinese community-dwelling older people. Geriatr. Nurs..

[4] Statistics Department, Ministry of Health and Welfare (2019). Elderly Status Survey. https://dep.mohw.gov.tw/dos/lp-1767-113.html.

[5] MacEntee M.I., Donnelly L.R. (2016). Oral health and the frailty syndrome. Periodontology 2000.

[6] Sánchez-García S., García-Peña C., Salvà A., Sánchez-Arenas R., Granados-García V., Cuadros-Moreno J., Velázquez-Olmedo L.B., Cárdenas-Bahena Á. (2017). Frailty in community-dwelling older adults: Association with adverse outcomes. Clin. Interv. Aging.

[7] Dörfer C., Benz C., Aida J., Campard G. (2017). The relationship of oral health with general health and NCDs: A brief review. Int. Dent. J..

[8] National Health Insurance Administration Ministry of Health and Welfare (2021). Medical Benefit. https://www.nhi.gov.tw/Content_List.aspx?n=076515B48BACD5A9&topn=CDA985A80C0DE710.

[9] Huang S.T. (2017). The Lost Part of the Elders with Systemic Frail: Oral Care. Long-Term Care Mag..

[10] World Health Organization (2018). Oral Health. http://www.who.int/news-room/factsheets/detail/oral-health.

[11] Tuuliainen E., Nihtilä A., Komulainen K., Nykänen I., Hartikainen S., Tiihonen M., Suominen A.L. (2020). The association of frailty with oral cleaning habits and oral hygiene among elderly home care clients. Scand. J. Caring Sci..

[12] Castrejón-Pérez R.C., Jiménez-Corona A., Bernabe E., Villa-Romero A.R., Arrivé E., Dartigues J.-F., Robledo L.M.G., Borges-Yáñez S.A. (2017). Oral Disease and 3-Year Incidence of Frailty in Mexican Older Adults. J. Gerontol. Ser. A Biol. Sci. Med. Sci..

[13] Castrejón-Pérez R., Borges-Yáñez S. (2014). Frailty from an Oral Health Point of View. J. Frailty Aging.

[14] Tanaka T., Takahashi K., Hirano H., Kikutani T., Watanabe Y., Ohara Y., Furuya H., Tetsuo T., Akishita M., Iijima K. (2018). Oral Frailty as a Risk Factor for Physical Frailty and Mortality in Community-Dwelling Elderly. J. Gerontol. Ser. A Biol. Sci. Med. Sci..

[15] Bakker M.H., Vissink A., Spoorenberg S.L.W., Wynia K., Visser A. (2020). Self-reported oral health problems and the ability to organize dental care of community-dwelling elderly aged ≥75 years. BMC Oral Health.

[16] Kamdem B., Seematter-Bagnoud L., Botrugno F., Santos-Eggimann B. (2017). Relationship between oral health and Fried’s frailty criteria in community-dwelling older persons. BMC Geriatr..

[17] Bassim C., Mayhew A.J., Ma J., Kanters D., Verschoor C.P., Griffith L.E., Raina P. (2020). Oral Health, Diet, and Frailty at Baseline of the Canadian Longitudinal Study on Aging. J. Am. Geriatr. Soc..

[18] Watanabe Y., Hirano H., Arai H., Morishita S., Ohara Y., Edahiro A., Murakami M., Pt H.S., Kikutani T., Suzuki T. (2016). Relationship Between Frailty and Oral Function in Community-Dwelling Elderly Adults. J. Am. Geriatr. Soc..

[19] Ramsay S.E., Papachristou E., Watt R., Tsakos G., Lennon L.T., Papacosta A.O., Moynihan P., Sayer A.A., Whincup P.H., Wannamethee S.G. (2018). Influence of Poor Oral Health on Physical Frailty: A Population-Based Cohort Study of Older British Men. J. Am. Geriatr. Soc..

[20] Avlund K., Schultz-Larsen K., Christiansen N., Holm-Pedersen P. (2011). Number of Teeth and Fatigue in Older Adults. J. Am. Geriatr. Soc..

[21] Castrejón-Pérez R.C., Borges-Yáñez S.A., Gutiérrez-Robledo L.M., Ávila-Funes J.A. (2012). Oral health conditions and frailty in Mexican community-dwelling elderly: A cross sectional analysis. BMC Public Health.

[22] Iwasaki M., Kimura Y., Sasiwongsaroj K., Kettratad-Pruksapong M., Suksudaj S., Ishimoto Y., Chang N.-Y., Sakamoto R., Matsubayashi K., Songpaisan Y. (2018). Association between objectively measured chewing ability and frailty: A cross-sectional study in central Thailand. Geriatr. Gerontol. Int..

[23] Pfeiffer E. (1975). A Short Portable Mental Status Questionnaire for the Assessment of Organic Brain Deficit in Elderly Patients. J. Am. Geriatr. Soc..

[24] Ensrud K.E., Ms S.K.E., Cawthon P.M., Fink H.A., Taylor B., Cauley J.A., Dam T.-T., Marshall L.M., Orwoll E., Cummings S.R. (2009). A Comparison of Frailty Indexes for the Prediction of Falls, Disability, Fractures, and Mortality in Older Men. J. Am. Geriatr. Soc..

[25] Ensrud K.E., Ewing S.K., Taylor B., Fink H.A., Cawthon P.M., Stone K.L., Hillier T.A., Cauley J.A., Hochberg M.C., Rodondi N. (2008). Comparison of 2 Frailty Indexes for Prediction of Falls, Disability, Fractures, and Death in Older Women. Arch. Intern. Med..

[26] Everaars B., Weening L., Jerković-Ćosić K., Schoonmade L., Bleijenberg N., De Wit N.J., Van Der Heijden G.J.M.G. (2020). Measurement properties of oral health assessments for non-dental healthcare professionals in older people: A systematic review. BMC Geriatr..

[27] Chalmers J., King P., Spencer A., Wright F., Carter K. (2005). The Oral Health Assessment Tool—Validity and reliability. Aust. Dent. J..

[28] Tavares M., Calabi K.A.L., Martin L.S. (2014). Systemic Diseases and Oral Health. Dent. Clin. N. Am..

[29] Zhang Y., Ge M., Zhao W., Hou L., Xia X., Liu X., Zuo Z., Zhao Y., Yue J., Dong B. (2020). Association Between Number of Teeth, Denture Use and Frailty: Findings from the West China Health and Aging Trend Study. J. Nutr. Health Aging.

[30] Nishimoto M., Tanaka T., Takahashi K., Unyaporn S., Fujisaki-Sueda-Sakai M., Yoshizawa Y., Iijima K. (2020). Oral frailty is associated with food satisfaction in community-dwelling older adults. Nihon Ronen Igakkai Zasshi.

[31] Tanaka T., Hirano H., Ohara Y., Nishimoto M., Iijima K. (2021). Oral Frailty Index-8 in the risk assessment of new-onset oral frailty and functional disability among community-dwelling older adults. Arch. Gerontol. Geriatr..

[32] Rapp L., Sourdet S., Vellas B., Lacoste-Ferré M.-H. (2017). Oral Health and the Frail Elderly. J. Frailty Aging.

[33] Dawes C., Wong D. (2019). Role of Saliva and Salivary Diagnostics in the Advancement of Oral Health. J. Dent. Res..

[34] Shimazaki Y., Nonoyama T., Tsushita K., Arai H., Matsushita K., Uchibori N. (2020). Oral hypofunction and its association with frailty in community-dwelling older people. Geriatr. Gerontol. Int..

[35] Komatsu R., Nagai K., Hasegawa Y., Okuda K., Okinaka Y., Wada Y., Tsuji S., Tamaki K., Kusunoki H., Kishimoto H. (2021). Association between Physical Frailty Subdomains and Oral Frailty in Community-Dwelling Older Adults. Int. J. Environ. Res. Public Health.

[36] Kugimiya Y., Watanabe Y., Ueda T., Motokawa K., Shirobe M., Igarashi K., Hoshino D., Takano T., Sakurai K., Taniguchi Y. (2020). Rate of oral frailty and oral hypofunction in rural community-dwelling older Japanese individuals. Gerodontology.

[37] Wilson D., Jackson T., Sapey E., Lord J. (2017). Frailty and sarcopenia: The potential role of an aged immune system. Ageing Res. Rev..

[38] Ballini A., Capodiferro S., Toia M., Cantore S., Favia G., De Frenza G., Grassi F. (2007). Evidence-Based Dentistry: What’s New?. Int. J. Med. Sci..

[39] Ballini A., Di Cosola M., Saini R., Benincasa C., Aiello E., Marrelli B., Saini S.R., Ceruso F., Nocini R., Topi S. (2021). A Comparison of Manual Nylon Bristle Toothbrushes versus Thermoplastic Elastomer Toothbrushes in Terms of Cleaning Efficacy and the Biological Potential Role on Gingival Health. Appl. Sci..

